# Oral treatment with the Chinese herbal supplements B307 enhances muscle endurance of ICR mice after exhaustive swimming via suppressing fatigue, oxidative stress, and inflammation

**DOI:** 10.1002/fsn3.1652

**Published:** 2020-05-19

**Authors:** Tai‐Yuan Chuang, Chia‐Ying Lien, Ya‐Chun Tsai, Kuei‐Fu Lin, Chih‐Hsiang Hsu, Wan‐Jhen Wu, Li‐Yu Su, Chen‐Wen Lu, Chung‐Hsin Wu

**Affiliations:** ^1^ School of Life Science National Taiwan Normal University Taipei City Taiwan; ^2^ Department of Athletics National Taiwan University Taipei City Taiwan; ^3^ Department of Physical Education National Tsing Hua University Hsinchu City Taiwan

**Keywords:** Chinese herbal supplements, exhaustive swimming, gastrocnemius muscles, ICR mice, inflammation, muscular endurance oxidative stress

## Abstract

Exhaustive exercise may damage muscles due to oxidative stress and inflammation and cause muscle fatigue and soreness. The study investigated the effects of Chinese herbal supplements (CHS) B307 on muscle endurance after exhaustive swimming (ES). Thirty‐two male ICR mice were randomly divided into 4 groups: Sham + ES, pretreatment of CHS B307 + ES (Pre + ES), post‐treatment of CHS B307 + ES (Post + ES), and dual treatment of CHS B307 + ES (Dual + ES). All mice were subjected to ES in the form of a forced swimming test. Then, we compared ES time (EST) as the index of muscular endurance. Also, we examined the fatigue, oxidative stress, inflammation, and damage in the muscle tissue among these groups by using immunohistochemistry (IHC), chemiluminescence, and biochemical analysis. Our results revealed that those mice of Pre + ES and Dual + ES groups had remarkably better EST than those mice of Sham + ES and Post + ES groups. Those mice with oral treatment of CHS B307(Pre + ES, Post + ES, and Dual + ES groups) showed significantly reduced leukocyte counts in the urine, and reduced levels of reactive oxygen species (ROS), neutrophils, and lactic acid in the blood than those mice of Sham + ES. In addition, those mice with oral treatment of CHS B307 (Pre + ES, Post + ES, and Dual + ES groups) showed significant alleviation of oxidative stress, inflammation, and damage in the muscle tissue than those mice of Sham + ES. Thus, we suggested that CHS B307 can be a functional sports supplement because it can enhance muscle endurance after exhaustive swimming via suppressing fatigue, oxidative stress, and inflammation.

## Graphical abstract

The study reported that Chinese herbal supplements B307 can be a functional sports supplement against exhaustive exercise.

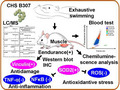

## 1 INTRODUCTION

Humans treated under acute endurance exercise often increase their oxygen consumption and reactive oxygen species (ROS), and cause muscle damage (Fuster‐Munoz et al., 2016; Popovic et al., 2012; Thirumalai, Viviyan Therasa, Elumalai, & David, 2011). It has been reported that ROS may play a crucial role in body defense systems (Powers & Jackson, 2008), but excessive ROS may cause many diseases such as diabetes, cancer, cardiovascular disease, and neurological disorders, and affect aging processes (Harman, 2006; Valko et al., 2007). Even though endogenous antioxidant capacity following exercise can partially attenuate oxidative stress by scavenging ROS (Kanter, Nolte, & Holloszy, 1993; Malaguti et al., 2009; Michailidis et al., 2007), high‐intensity exercise may generate a lot of ROS that far more than our antioxidant capacity (Sies & Jones, 2007). Many sports science studies have indicated that skeletal muscle fibers exposed to a high concentration of ROS may lead to impaired muscle force production and fatigue (Andrade, Reid, Allen, & Westerblad, 1998; Reid, Stokić, Koch, Khawli, & Leis, 1994; Shindoh, DiMarco, Thomas, Manubay, & Supinski, 1990).

In the case of high‐intensity exercise with limited recovery periods, athletes often show athletic performance decline and muscle fatigability increase subsequently (Margonis et al., 2007). It was reported that excessive exercise may affect the balance of hormone adjustment, and then cause oxidative stress and muscle damage in rats (Zhou et al., 2019). Exhaustive exercise testing with forced swimming that has been widely applied to examine physiological damage after high‐intensity exercise (dos Reis, Martins, de Araujo, & Gobatto, 2018). In exhaustive exercise testing with forced swimming, exercise intensity can be indirectly graded by the addition of a weight load attached to the body or tail to decrease the time until exhaustion (McArdle & Montoye, 1966).

Unlike health‐promoting exercise, athletes must undergo severe training daily to improve their athletic performance; therefore, it is essential to develop an appropriate sports supplement that can protect muscles against oxidative stress, inflammation, and damage. It was reported that *Danggui Buxue Tang* has a favorable antifatigue effect in male mice after forced swimming (Miao et al., 2018). To our knowledge, however, the antioxidant, anti‐inflammatory, and muscle‐relieving effects of Chinese herbal supplements (CHS) after forced swim tests have rarely been elucidated. As suggested in our previous studies, we have reported that CHS B307 can effectively alleviate oxidative stress, inflammation, and damage in the myocardial tissue (Lin, Wang, & Hsu, 2015; Lien et al., 2015). The main herbal ingredients in CHS B307 were ginseng (*Panax ginseng Radix*), schizandra (*Schizandrae Fructus*), ophiopogon (*Ophiopogonis Tuber*), and danshen (*Salviae Miltiorrhizae Radix*) that also been reported in antioxidant, anti‐inflammatory, and muscle‐relieving effects (Ahuja, Goswami, & Adhikari, 1992; Chen et al., 2003; Lee, Song, Sohn, & Shin, 2012; Lin, Zhang, Moldzio, & Rausch, 2007; Sieveking et al., 2005; Tam et al., 2009; Xu et al., 2007). We believe that CHS B307 may be an appropriate sports supplement to protect muscle against oxidative stress and inflammation for athletes training under a high exercise intensity. The results of this study may provide concrete scientific evidence concerning that CHS B307 could be an ideal candidate for a sports supplement because of its antifatigue, antioxidation, anti‐inflammation, and antidamage effects in skeletal muscles.

## 2 MATERIALS AND METHODS

### 2.1 Animal preparation

Thirty‐two male ICR mice were purchased from BioLASCO Taiwan Yi‐Lan Breeding Center (fully accredited by AAALAC International). In accordance with the Institutional Guidelines of the Animal Care and Use Committee of National Taiwan Normal University (NTNU), the mice were maintained in the animal facility of NTNU under specific pathogen‐free conditions. All animal experiments were approved by the Institutional Animal Care and Use Committee of our university (Protocol number: NTNU Animal Experiments No. 108011). All the mice were housed in an environment with a constant temperature of 22°C ± 2°C and subjected to a 12‐hr light/dark cycle, and the mice had ad libitum access to water and food.

### 2.2 Study design

The study assessed the effects of CHS B307 on muscle endurance and recovery after ES in ICR mice. All mice were subjected to forced ES executed under a forced swimming test. A total of 32 male ICR mice were randomly divided into four groups: Sham + ES, CHS B307 pretreatment + ES (Pre + ES), CHS B307 post‐treatment + ES (Post + ES), and CHS B307 dual treatment + ES (Dual + ES). The Pre + ES mice were orally given CHS B307 treatment before ES, Post + ES mice were orally given CHS B307 treatment after ES, Dual + ES mice were orally given CHS B307 treatment both before and after ES, and the Sham + ES group was given a normal diet and drinking water before and after ES. The experiment was approved by the Institutional Animal Care and Use Committee of NTNU (protocol number NTNU Animal Experiments No. 108011).

### 2.3 ES experiment design

As suggested in (Li & Chen, 2018), a weight‐loaded ES procedure was carried out in a swimming tank (50 × 50 × 50 cm^3^) with 30‐cm‐deep water maintained at 25 ± 3°C for 1 hr after the oral administration of CHS 307. Then, mice were removed from the pool, dried with a paper towel, and returned to their original cages. The pool water was replaced after each session. ES was determined by observing the loss of coordinated movements and failure to swim. ES time was recorded immediately when mice were completely exhausted and failed to return to the surface to breathe within 5 s.

### 2.4 Oral treatment of CHS B307

CHS B307 is a commercially available nutritional supplements (Sun‐Ten Pharmaceutical Company). The Pre + ES, Post + ES, and Dual + ES groups were orally treated with CHS B307 extract. In this study, we used a gavage tube to orally administer CHS B307 extract (50 mg/ml; the pH value was approximately 7.0) and its vehicle (dimethyl sulfoxide). Doses were adjusted according to the individual weight of the mouse. As suggested in our previous study (Lin et al., 2015; Lien et al., 2015), the feeding dose of CHS B307 was 50 mg/kg BW, and the feeding frequency was 4 times for the Pre + ES and Post + ES mice and 8 times for the Dual + ES mice at intervals of 12 hr. The dosage and administration of CHS B307 extract were much lower than the median lethal dose (LD_50_). The sham group was orally treated with the vehicle.

### 2.5 Chromatographic fingerprint analysis

**Figure 1:**
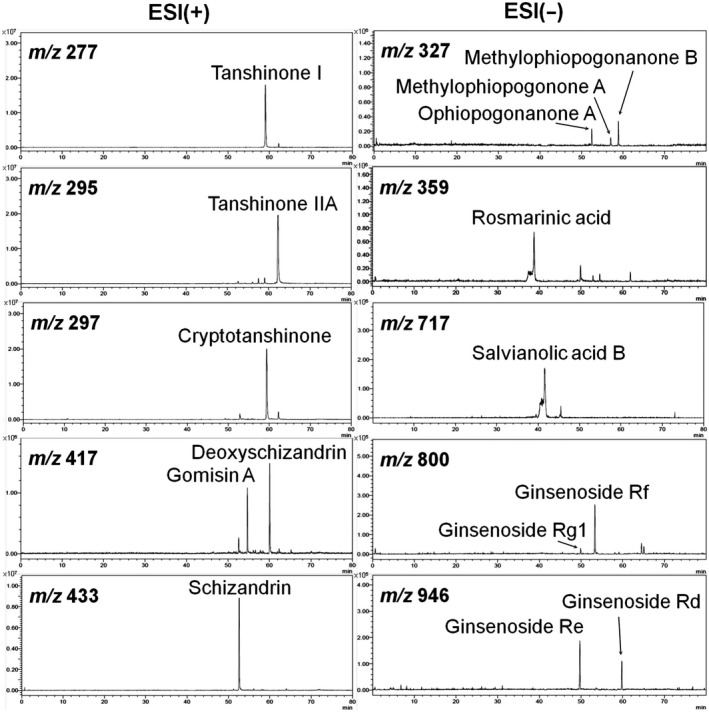
Chromatographic fingerprint analysis for the CHS B307 that was conducted by using LC/MS analysis. Bioactive marker substances for *Ginseng Radix*: Ginsenoside Rg1, Ginsenoside Re, Ginsenoside Rf, Ginsenoside Rd; *Schizandrae Fructus*: Schizandrin, Gomisin A, Deoxyschizandrin; *Ophiopogonis Tuber*: Ophiopogonanone A, Methylophiopogonone A, Methylophiopogonanone B; *Salviae Miltiorrhizae Radix*: rosmarinic acid, salvianolic acid B, cryptotanshinone, tanshinone I, and tanshinone IIA. Bioactive marker substances from ingredients of the CHS B307 were qualitatively determined within 80 min under the selected LC/MS condition. Abbreviations: AU, arbitrary perfusion units; LC/MS, liquid chromatography–mass spectrometry; ESI, electrospray ionization; *m*/*z*, mass‐to‐charge ratio

All CHS B307 (supplied by Sun‐Ten Pharmaceutical Company) were solubilized in distilled H_2_O/MeOH and then analyzed using liquid chromatography–mass spectrometry (LC/MS) analysis. Fifteen bioactive marker substances were qualitatively determined within 80 min under a selected LC/MS condition, as shown in Figure 1. As suggested in our previous study (Lien et al., 2015), the LC/MS analytical system consisted of a Shimadzu LC‐20AD UFLC system linked with a LCMS‐8040 triple quadrupole mass spectrometer. The UFLC condition was set as follows: gradient elution by the mixture of mobile phases A (0.1% formic acid and 1 g/L solution of ammonium acetate in H2O) and B (0.1% formic acid and 1 g/L solution of ammonium acetate in MeOH) at minutes 0–40 with the ratio of 100%–70% A and 0%–30% B; at minutes 40–70 with the ratio of 70%–0% A and 30%–100% B; at minutes 70–70.1 with the ratio of 0%–100% A and 100%–0% B; and at minutes 70.1–80 with the ratio of 100% A and 0% B. The flow rate was 0.4 ml/min; the column temperature was kept at 40°C; the injection volume was 20 μl; and the analytical column was a Shimadzu Shim‐pack XR‐ODS II column (2.2 μm, 2 × 100 mm, Shimadzu). Dual ion modes [electrospray ionization, ESI(+) and ESI(−)] were used in MS detection, and the transmission of [M + H]+and [M−H] − was set as the optimum condition. The MS detection was set as a full scan range (100–1,200 amu); the interface voltages were set at 4.5 kV for ESI(+) and −3.5 kV for ESI(−). Nitrogen as a nebulizing and drying gas, the flow was at 3.0 and 10 L/min, respectively. Argon as a CID gas was set at 230 kPa. DL temperature was at 150°C, whereas heat block temperature was at 400°C.

### 2.6 Urine and blood tests

After the final ES procedure, mice were immediately sacrificed under mild anesthesia. Urine was collected from the bladder, and blood was collected from the abdominal aorta into centrifuge tubes using a heparinized syringe. The urine samples were analyzed using 10 parameter urinalysis reagent strips for urine tests (Medisave Ltd.). The blood samples were analyzed using a blood smear, as is common practice to evaluate the health status of an animal. The blood samples were taken in a blood sampling tube with heparin (Terumo Inc.) and centrifuged for 10 min at 1,000 *g* at 4°C to separate the plasma, which was stored at −80°C until analysis. The plasma samples were analyzed for lactate acid using an assay kit (Cayman Chemical). To determine blood ROS levels, lucigenin‐ and luminol‐amplified chemiluminescence (CL) methods were used to measure O2^·−^ and H_2_O_2_ activity. The lucigenin‐enhanced CL method is reliable for ROS assays. A heparinized sample (0.2 ml) of whole blood was taken from the left femoral artery of each mouse. ROS blood levels were measured using a CL analyzer (CLA‐ID3 chemiluminescence analyzer; Tohoku Electronic Industrial Co., Ltd.) after the addition of 1.0 ml of 0.1 mM lucigenin in phosphate‐buffered saline (pH 7.4) to the samples. The assay was duplicated for each sample, and total CL counts in 600 s were calculated by integrating the area under the curve.

### 2.7 Immunohistochemistry (IHC) analysis

ICR mice were anesthetized and then cardiac‐perfused with phosphate‐buffered saline containing 4% formaldehyde (EM grade glutaraldehyde solution, Sigma‐Aldrich St. Louis, MU, United States). Gastrocnemius muscle tissue was then removed and fixed with 4% formaldehyde (EM grade). The muscle specimens were embedded in paraffin and cut into tissue sections with a thickness of 5 μm. The tissue sections were mounted on slides for histological and muscle immunohistochemistry (IHC) analysis. Using the heat‐induced epitope retrieval method, the sections were separately stained at room temperature for 1 hr with antibodies of superoxide dismutase 2 (SOD2; Cat. #13141, Cell Signaling Technology Inc.), tumor necrosis factor alpha (TNF‐α) (Cat. #11948, Cell Signaling Technology Inc.), nuclear factor kappa‐light‐chain‐enhancer of activated B cells (NFκB) (Cat. #3034, Cell Signaling Technology Inc.), and vinculin (Cat. #13901, Cell Signaling Technology Inc.) immunostaining controls for each antibody. Serial 5‐μm cross sections were treated with the unanimous staining protocol. Immunostaining detection was executed using incubation with biotinylated secondary antibodies (NovolinkTM polymer detection system l) at room temperature for 30 min and then by incubation with avidin–biotin–HRP complex (NovolinkTM polymer detection system l) for an additional 30 min. Immunostaining visualization was performed with DAB Chromogen (NovolinkTM polymer detection system l) and counterstained with hematoxylin (NovolinkTM polymer detection system l) following the supplier's protocol.

### 2.8 Western blot analysis

The removed gastrocnemius muscle tissue was kept in a buffer solution to maintain its pH for a Western blot analysis. Skeletal muscle protein was subjected to sodium dodecyl sulfate–polyacrylamide gel electrophoresis and transferred to a polyvinylidene difluoride membrane. We used SOD2 antibodies (Cat. #13141, Cell Signaling Technology Inc.) to identify expression levels of antioxidative proteins in the skeletal muscle tissue by means of a horseradish peroxidase‐linked secondary antibody. In addition, enhanced chemiluminescence Western blotting detection reagents (GE Healthcare Life Sciences,) were used to make immunoreactive bands perceptible. An ImageQuant LAS‐4000 biomolecular imager (GE Healthcare Life Sciences) was used to detect chemiluminescence. Image J software (version 1.48t, Rasband, W.S., ImageJ, U. S. National Institutes of Health, Bethesda, Maryland, USA) was used to count densitometric assessments of the bands.

### 2.9 Statistical analysis

The data in Figures 3, 4, and 6 were obtained from at least three independent experiments. Values for the data were expressed as mean ± standard error of the mean (*SEM*). Differences among the ICR mice groups were evaluated using a two‐way analysis of variance (ANOVA). If a significant *F*‐value was observed, the Student–Newman–Keuls multiple comparisons post‐test was conducted to determine where differences existed. *p* values of < .05 were considered significant.

## 3 RESULTS

### 3.1 Chromatographic fingerprint of CHS B307

Chromatographic fingerprint analysis of CHS B307 is shown in Figure 1. Ginseng (*panax ginseng Radix*), schizandra (*schizandrae Fructus*), ophiopogon (*ophiopogonis Tuber*), and danshen (*salviae miltiorrhizae Radix*) are the main herbal ingredients in CHS B307. A bioactive marker substance for *ginseng Radix* is ginsenoside Rb1; marker substances for *schizandrae Fructus* are schizandrin and gomisin A; a marker substance for *ophiopogonis Tuber* is methylophiopogonanone B; and marker substances for *salviae miltiorrhizae Radix* are rosmarinic acid, salvianolic acid B, and tanshinone IIA.

### 3.2 Effects of oral CHS B307 treatment in body weight and EST

**Figure 2:**
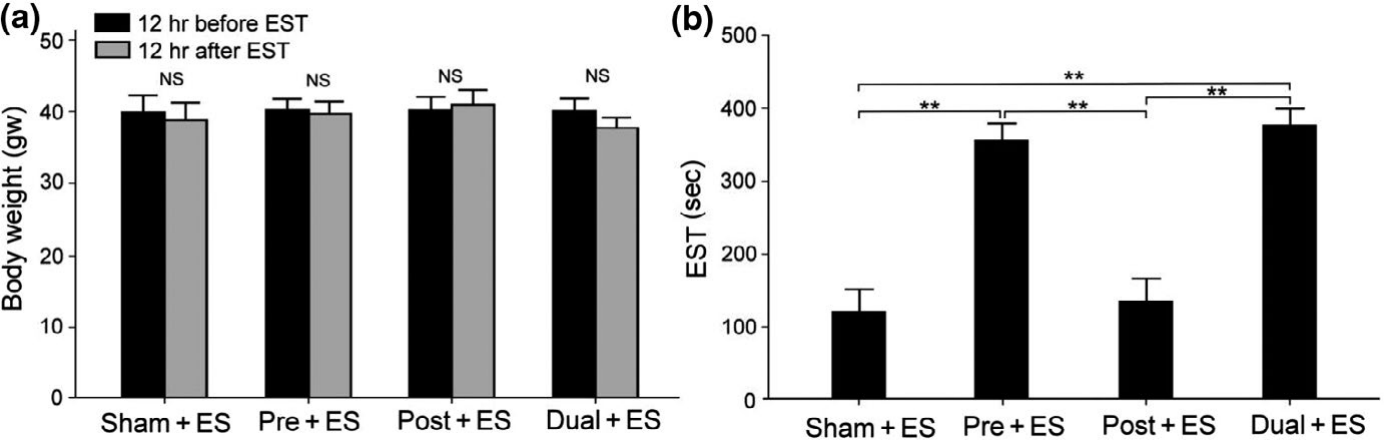
Comparisons of the body weight and exhaustive swimming time (EST) between those mice with and without CHS B307 treatments. (a) Statistical comparison of quantified body weight 12 hr before and after the ES test. There are no any differences (paired *t* test, *p *> .05) among the Sham + ES, Pre + ES, Post + ES, and Dual + ES groups. (b) Statistical comparison of quantified EST shows that the ESTs of Pre + ES and Dual + ES mice were significantly longer than those of Sham + ES and Post + ES mice (**p < .01, one‐way ANOVA followed by Student–Newman–Keuls multiple comparison post‐test). All data are shown as mean ± *SEM*. Eight mice were conducted for each treatment

CHS B307 is related to cardiac function and skeletal muscle strength in mice (Lin et al., 2015; Lien et al., 2015). We sought to determine whether any change in muscle endurance to ES occurred with oral CHS B307 treatment. We measured the body weight and ES time of ICR mice after oral CHS B307 treatment. After ES, no significant difference was observed in body weight in any group (*p *> .05, Figure 2a). However, as presented in Figure 2b, the Pre + ES and Dual + ES groups had ES times 250% longer than those of the Sham + ES and Post + ES groups (*p *< .01, Figure 2b). The results suggest that CHS B307 pretreatment promoted muscle endurance in the mice.

### 3.3 Effects of oral CHS B307 treatment in the urine and the blood after ES

**Figure 3:**
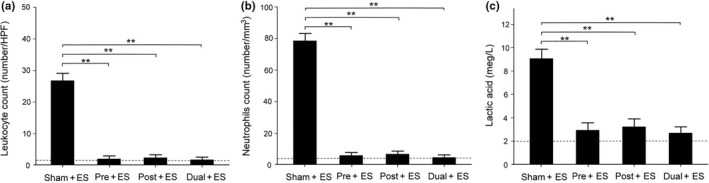
Comparisons of the urine and blood tests between those mice with and without CHS B307 treatments. A, Statistical comparison of quantified leukocyte counts in urine shows that leukocyte counts of Pre + ES, Post + ES, and Dual + ES mice were significantly lower than those of Sham + ES mice (*p *< .01). B, Statistical comparison of quantified neutrophil counts in the blood shows that neutrophils counts in the Pre + ES, Post + ES, and Dual + ES mice were significantly lower than those of Sham + ES mice (*p *< .01). C, Statistical comparison of quantified lactic acid in the blood shows that lactic acid levels in the Pre + ES, Post + ES, and Dual + ES mice were significantly lower than those of Sham + ES mice (*p *< .01). The dashed lines represent averaged values in normal mice. All data are shown as mean ± *SEM* (**p < .01, two‐way ANOVA followed by Student–Newman–Keuls multiple comparison post‐test). Eight mice were conducted for each treatment

We examined the effects of oral CHS B307 treatment in inflammatory factors in urine and blood. Figure 3a presents a comparison of leukocyte counts in urine among the groups. Leukocyte counts in the urine of the Sham + ES mice were significantly increased after ES, whereas the leukocyte counts of the Pre + ES, Post + ES, and Dual + ES mice were significantly decreased (*p *< .01, Figure 3a). We also compared neutrophil counts in blood samples (Figure 3b). Compared with normal mice, neutrophil counts for the Sham + ES mice were significantly increased after ES, and neutrophil counts for the Pre + ES, Post + ES, and Dual + ES mice were significantly decreased (*p *< .01, Figure 3b). To examine the effects of oral CHS B307 treatment on muscle fatigue, we compared lactic acid levels in the blood of the mice (Figure 3c). Lactic acid levels of the Sham + ES mice were significantly increased after ES, whereas those of the other groups were significantly decreased (*p *< .01, Figure 3c).

### 3.4 Effects of oral CHS B307 treatment on antioxidant stress in muscle tissue after ES

**Figure 4:**
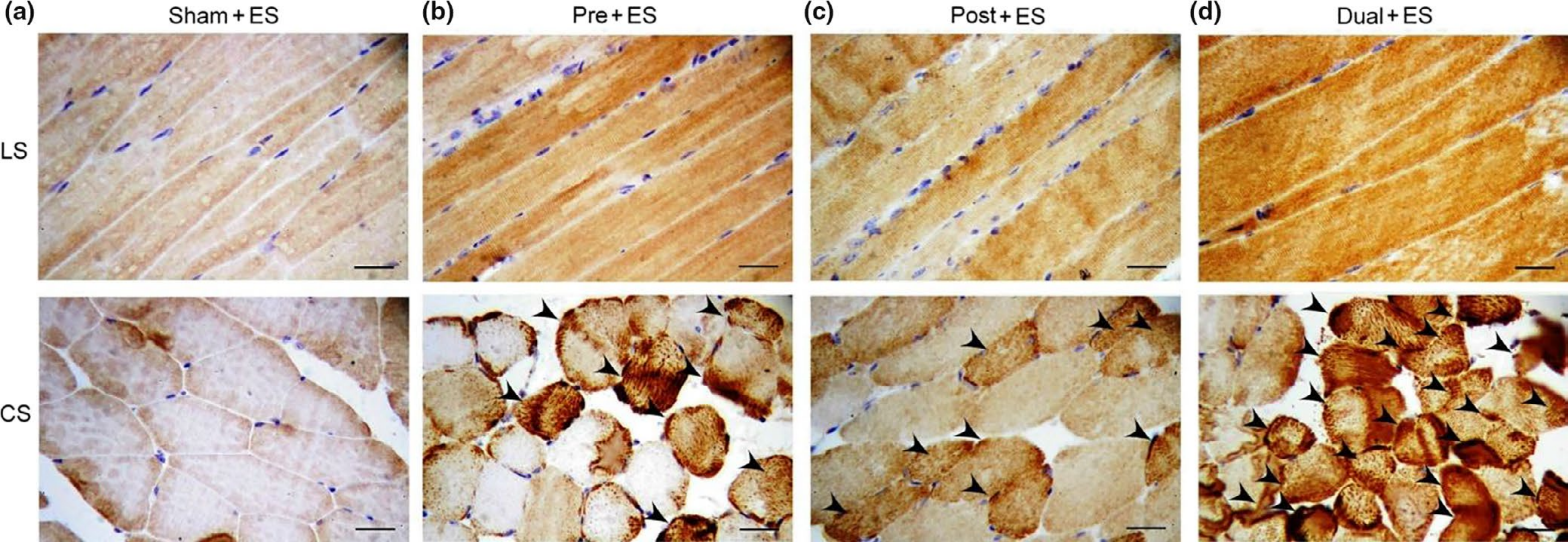
Representative IHC expressions of antioxidant stress‐related SOD2 in muscle tissue between the mice with and without CHS B307 treatments. IHC staining of longitudinal sections (LS) and cross sections (CS) of gastrocnemius muscle tissue shows that SOD2 expressions (dark block that marked with arrows) in muscle tissue of Pre + ES, Post + ES, and Dual + ES mice were obviously higher than those in Sham + ES mice. Scale bar = 30 μm

**Figure 5:**
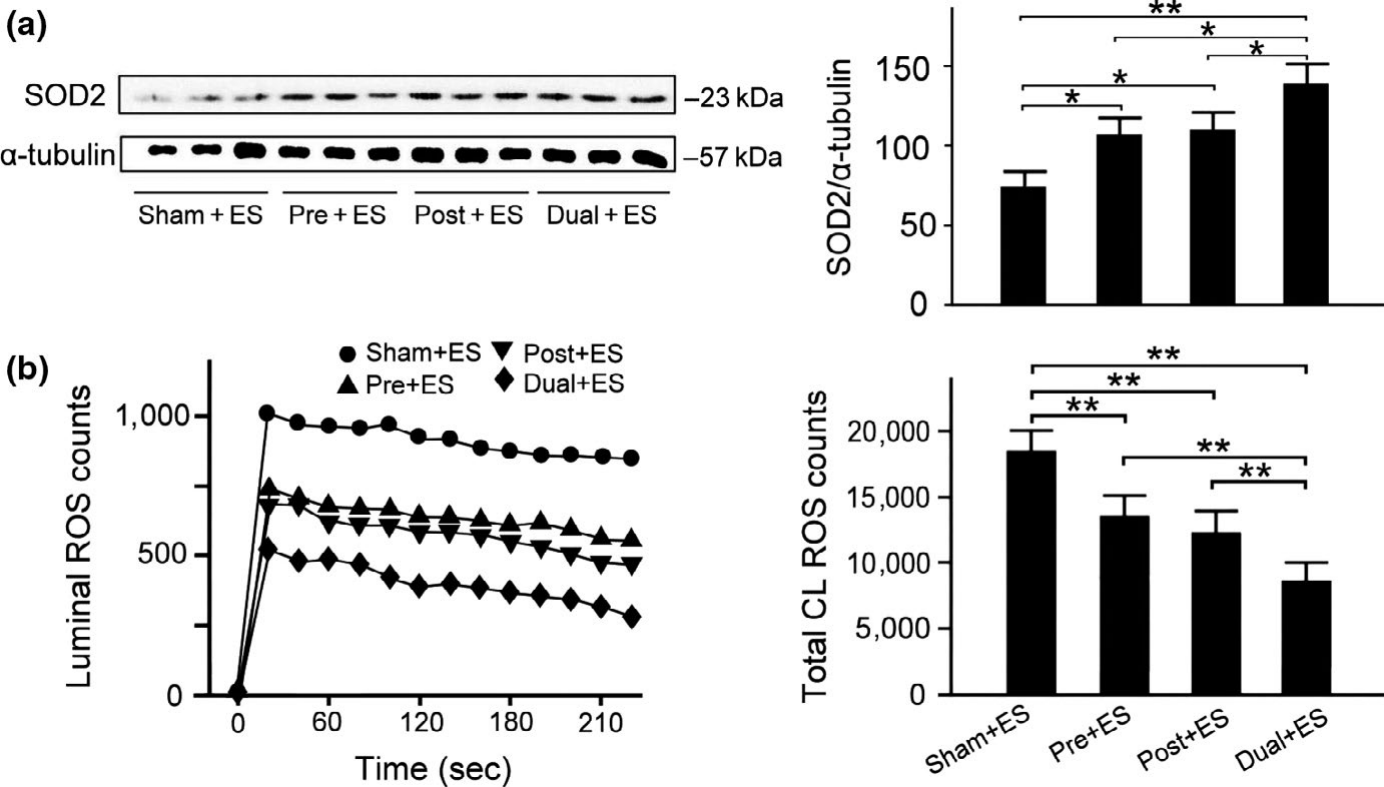
Comparisons of antioxidant stress‐related SOD2 in muscle tissue, and the reactive oxygen species (ROS) in the blood between those mice with and without CHS B307 treatments. A, Western blotting expressions of gastrocnemius muscle tissue shows that SOD2 expressions in muscle tissue of Pre + ES, Post + ES, and Dual + ES mice were significantly greater than those of Sham + ES mice (*p* < .01–0.05). B, Blood ROS expressions through chemiluminescence analysis show that ROS expressions in the blood of Pre + ES, Post + ES, and Dual + ES mice were significantly lower than those of Sham + ES mice (*p* < .01). All data are shown as mean ± *SEM* (**p < .01, *p < .05, two‐way ANOVA followed by Student–Newman–Keuls multiple comparison post‐test). Experiments were repeated 3 times for each treatment

To examine the effects of oral CHS B307 treatment on oxidative stress in muscle tissue after ES, we evaluated expression levels of SOD2 in gastrocnemius muscles using IHC and biochemical analysis because SODs are enzymes that reflect antioxidative capacity. As indicated in Figure 4, SOD2 expression in the Pre + ES, Post + ES, and Dual + ES mice was markedly higher than in the Sham + ES mice. Western blotting (Figure 5a) revealed SOD2 expression in the treated mice was significantly greater than that in the Sham + ES mice (*p*< .01–.05, Figure 5a), Notably, SOD2 expression in the Dual + ES mice was significantly higher than in the Pre + ES and Post + ES mice (*p *< .05, Figure 5a). We further examined blood ROS expression among the 4 groups through chemiluminescence analysis, because SOD2 can clear mitochondrial ROS. Figure 5b shows that the expression of blood ROS in the treated mice was lower than in the Sham + ES mice, and the expression of blood ROS in the Dual + ES mice was lower than in the Pre + ES and Post + ES groups. Figure 5b shows that quantified blood ROS expression in gastrocnemius muscle tissue of the Pre + ES, Post + ES, and Dual + ES mice was significantly lower than in the Sham + ES mice (*p *< .01–0.05, Figure 5b). Notably, quantified blood ROS expression in the Dual + ES mice was significantly lower than in the Pre + ES and Post + ES groups (*p *< .01, Figure 5b).

### 3.5 Effects of oral CHS B307 treatment on inflammation in muscle tissue after ES

**Figure 6:**
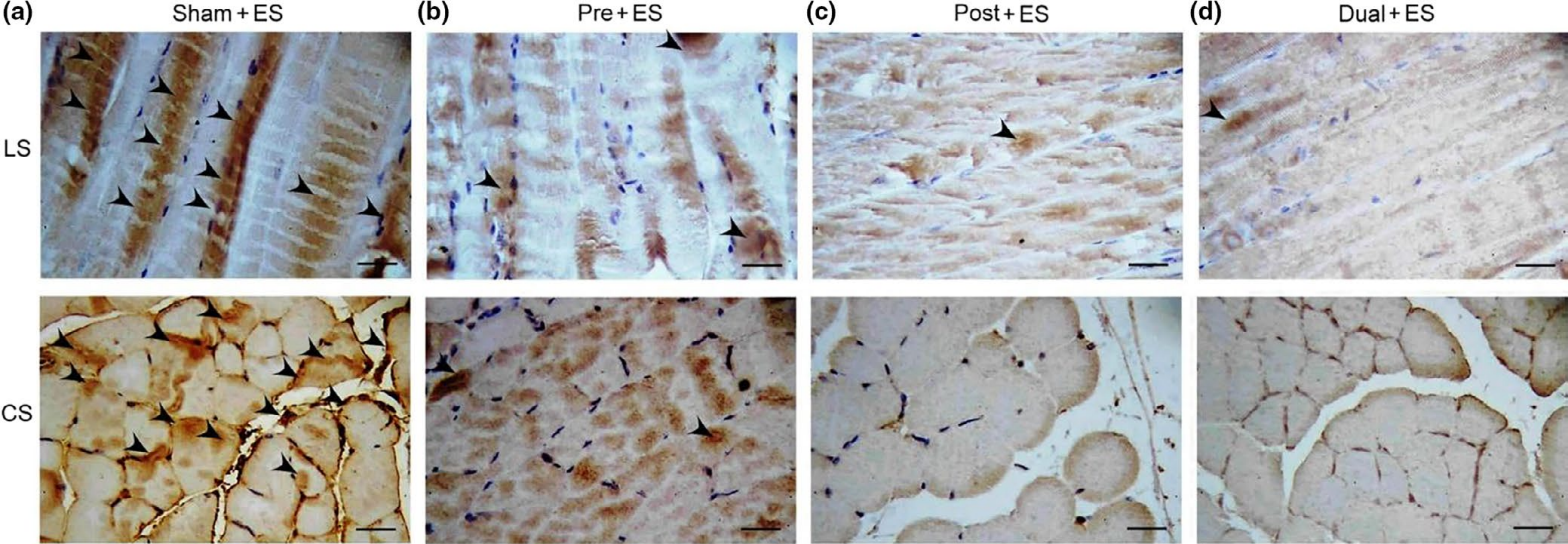
Representative IHC expressions of inflammation‐related TNF‐α in muscle tissue between those mice with and without CHS B307 treatments. IHC staining of longitudinal sections (LS) and cross sections (CS) of gastrocnemius muscle tissue shows that TNF‐α expressions (dark block that marked with arrows) in muscle tissue of Pre + ES, Post + ES, and Dual + ES mice were obviously weaker than those in Sham + ES mice. Scale bar = 30 μm

**Figure 7:**
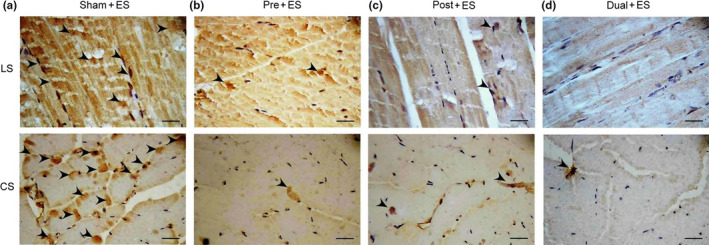
Representative IHC expressions of inflammation‐related NFκB in gastrocnemius muscle tissue between those mice with and without CHS B307 treatments. IHC staining of longitudinal sections (LS) and cross sections (CS) of gastrocnemius muscle tissue shows that NFκB expressions (dark block that marked with arrows) in muscle tissue of Pre + ES, Post + ES, and Dual + ES mice were obviously weaker than those in Sham + ES mice. Bar scale = 30 μm

To examine the effects of oral CHS B307 treatment on muscle tissue inflammation after ES, we compared expressions of TNF‐α and NFκB in the gastrocnemius muscles of mice after ES. As revealed by IHC staining (Figure 6), TNF‐α expression in the treated mice was markedly weaker than in the Sham + ES mice. Furthermore, as Figure 7 shows, NFκB expression in the treatment groups was obviously weaker than in the Sham + ES group.

### 3.6 Effects of oral CHS B307 treatment on damage in muscle tissue after ES

**Figure 8:**
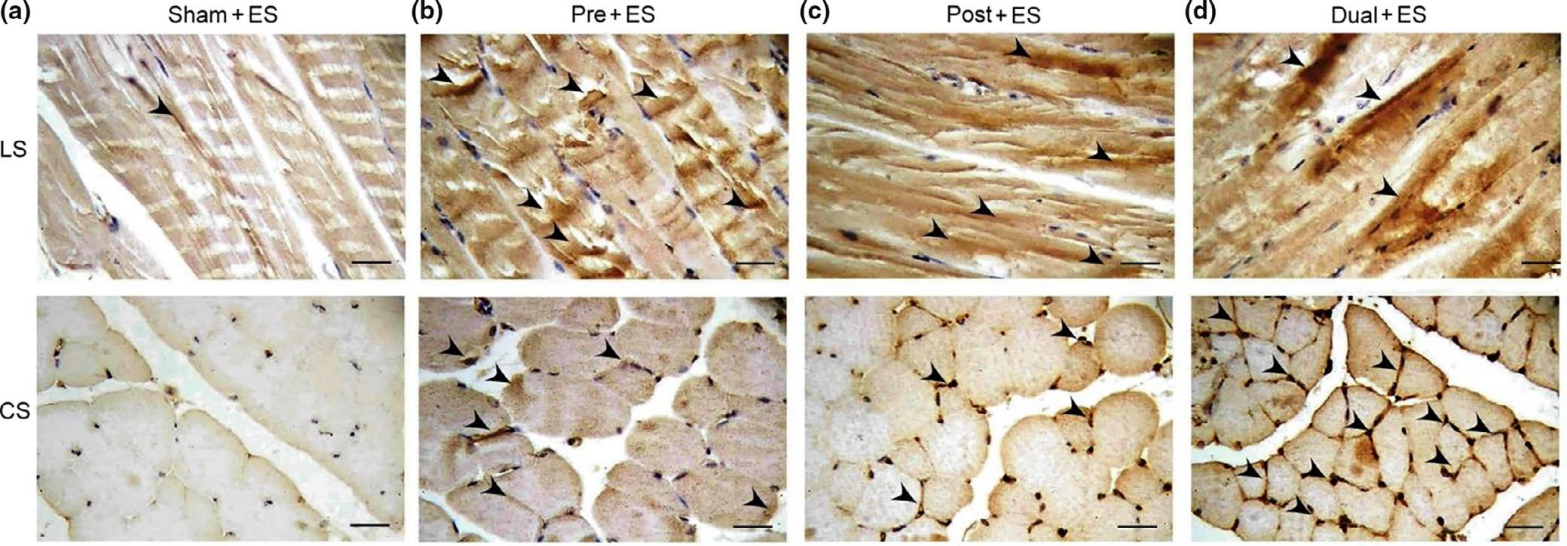
Representative IHC expressions of membrane‐cytoskeletal vinculin in gastrocnemius muscle tissue between those mice with and without CHS B307 treatments. IHC staining of longitudinal sections (LS) and cross sections (CS) of gastrocnemius muscle tissue shows that vinculin expressions (dark block that marked with arrows) in muscle tissue of Pre + ES, Post + ES, and Dual + ES mice were obviously higher than those in Sham + ES mice. Scale bar = 30 μm

In mammalian cells, vinculin plays a role in muscle repair systems. Thus, we compared vinculin expression in gastrocnemius muscle tissue among the groups through IHC staining. As shown in Figure 8, vinculin expression in the treated mice was obviously stronger than in the sham‐treated mice after ES. The results demonstrate that CHS B307 modulated focal adhesion structure in skeletal muscle after ES.

## 4 DISCUSSION

Here, we reported CHS B307 may be considered as a potential sports supplement for exhaustive exercise. The main herbal ingredients in CHS B307 were ginseng (*Panax ginseng Radix*), schizandra (*Schizandrae Fructus*), ophiopogon (*Ophiopogonis Tuber*), and danshen (*Salviae Miltiorrhizae Radix*). Many studies have reported that ginseng has properties of antioxidant (Chen et al., 2003) and anti‐inflammatory (Lee et al., 2012; Lin et al., 2007). Others have reported that ginseng can improve alertness and fatigue resistance through cortisol stimulation (Ahuja et al., 1992). Danshen can alleviate heart disease and ameliorate the effects of atherosclerosis in humans (Sieveking et al., 2005; Xu et al., 2007) and rodents (Tam et al., 2009). As suggested in a review paper (Sellami et al., 2018), ginseng has been used as an endurance performance enhancer. Alkaloid supplements can improve athletic performance for intense sprinting and cycling exercises. Some other alkaloids such as green tea extracts can increase body mass and improve muscle composition in athletes. In this study, our results show that those mice with CHS B307 treatment does not seem to change the body weight after ES (Figure 2a), but can promote muscle endurance because of longer EST than those mice without CHS B307 treatment (Figure 2b).

In the urine and blood, leukocyte and neutrophils can mediate acute inflammatory tissue damage because they can act against infectious microbes and produce ROS to kill invading microorganisms (Smith, 1994). Our results reported that those mice with CHS B307 treatment can alleviate acute inflammatory tissue damage after ES because of significantly reduced counts of leukocyte and neutrophils than those mice without CHS B307 treatment (Figure 3a‐b). There was also reported that acute exercise may activate the NFκB‐TNFα inflammatory signaling pathway in rat skeletal muscles (Ji, Gomez‐Cabrera, Steinhafel, & Vina, 2004). TNF‐α has been associated with muscle inflammation that may be mediated by ROS and NFκB, both of which upregulate ubiquitin/proteasome activity (Thoma & Lightfoot, 2018). Thus, TNF‐α and NFκB may play key roles in muscle inflammation after ES. Our results demonstrate that those mice with CHS B307 treatment can alleviate muscle inflammation after ES because of significantly reduced expressions of TNF‐α and NFκB than those mice without CHS B307 treatment (Figures 6 and 7). After exhaustive exercise, neutrophil activation can result in the overproduction of ROS, which contributes to muscle injury and leads to oxidative stress (Pizza, Peterson, Baas, & Koh, 2005; Suzuki et al., 1996). We have observed that those mice can significantly increase the ROS levels after ES, while CHS B307 treatment may significantly decrease the ROS levels after ES (Figure 5b). Mitochondrial SOD2 plays an essential role in endogenous antioxidant capacity, which can inhibit oxidative stress. Our IHC and Western blotting evidences demonstrated that those mice with CHS B307 treatment can enhance their endogenous antioxidant capacity after ES because of significant increased expressions of SOD2 in gastrocnemius muscle tissue than those mice without CHS B307 treatment (Figures 4, 5a). The above results clearly support CHS B307 could be an ideal sports supplement because of alleviating exercise‐induced inflammation and oxidative stress in muscle tissue after ES.

During intense exercise, lactic acid is produced in muscles and then accumulated, which can lead to muscle fatigue and pain. Our findings indicate that those mice with CHS B307 treatment may alleviate muscle fatigue and pain after ES because of significantly reduced levels of lactic acid than those mice without CHS B307 treatment (Figure 3c). In mammalian cells, vinculin is a membrane‐cytoskeletal protein involved in the linkage of adhesion molecules to actin cytoskeleton (Goldmann & Ingber, 2002). After exhaustive exercise, muscle injuries are often occurred and accompanied by decreasing expressions of vinculin in the membrane of muscle cells. Our study demonstrates that those mice after ES may decrease expressions of vinculin in the membrane of muscle cells, but CHS B307 treatment can alleviate muscle injuries because of significantly increased expressions of vinculin in the muscle tissue (Figure 8). In other words, pretreatment of CHS‐B307 should have protective effects on muscle tissue of ICR mice.

## 5 CONCLUSIONS

Our studies demonstrate that those mice with oral treatment of CHS B307 can effectively enhance muscle endurance by prolonging EST. For those mice after ES, CHS B307 treatment may alleviate ES‐induced inflammation and oxidative stress by reducing counts of leukocyte and neutrophils in the blood and urine, decreasing ROS levels in the blood, and suppressing expressions of inflammation‐related TNF‐α and NFκB, but increasing expressions of antioxidant related SOD2 in muscle tissue. In addition, CHS B307 treatment may mitigate muscle fatigue and injuries for those mice after ES because of decreasing expressions of fatigue‐related lactic acid, but increasing expressions of membrane‐ cytoskeletal vinculin. Thus, we suggested that CHS B307 can be an ideal sports supplement for exhaustive exercise because of antioxidant, anti‐inflammation, antifatigue, and antidamage capacities in skeletal muscles after ES.

## CONFLICT OF INTERESTS

The authors declare no conflicts of interest in this work.

## ETHICAL REVIEW

This study was approved by the Institutional Animal Care and Use Committee of National Taiwan Normal University (Protocol number: NTNU Animal Experiments No. 108011).
